# Hydrothermal Synthesis of Zinc Stannate Nanoparticles for the Electrochemical Detection of Organophosphate Pesticide—Parathion-Ethyl

**DOI:** 10.3390/s25092837

**Published:** 2025-04-30

**Authors:** Loganathan Vagismathi, Sea-Fue Wang

**Affiliations:** Department of Materials and Mineral Resources Engineering, National Taipei University of Technology, Taipei 106, Taiwan; t113a09413@ntut.edu.tw

**Keywords:** zinc stannate, parathion-ethyl, electrochemical sensor, Zn_2_SnO_4_/SnO_2_, pesticide detection

## Abstract

This work focuses on developing a Zn_2_SnO_4_-based electrochemical sensor for detecting parathion-ethyl (EP), a toxic organophosphorus pesticide. Monitoring such hazardous compounds is essential to ensure environmental and food safety. Zn_2_SnO_4_, known for its excellent electrical conductivity, catalytic activity, simple synthesis process, and eco-friendly nature, was utilized as an electrode material to enhance the detection of EP. Zn_2_SnO_4_ was synthesized via a hydrothermal method and characterized using XRD to confirm its crystalline structure. Zn_2_SnO_4_ was subsequently modified onto a glassy carbon electrode (GCE), enabling the study of its electrochemical properties and interaction with EP. River water and carrot samples were collected, pretreated, and analyzed for EP detection to evaluate real-world applicability. Electrochemical detection of EP using differential pulse voltammetry (DPV) showed a linear response in the concentration range of **0.01–78.4** μM, with a detection limit of 0.0059 µM. The sensor demonstrated excellent repeatability and selectivity in the presence of potential interferents. Real sample analysis confirmed the sensor’s effectiveness, achieving satisfactory recovery rates in river water and carrot samples. The high surface area and conductivity of Zn_2_SnO_4_ significantly enhanced the electrochemical response, validating its potential for reliable EP detection in environmental and agricultural samples.

## 1. Introduction

The widespread use of insecticides, pesticides, and acaricides is essential for protecting food crops from damage caused by insects and pests. Pesticides are classified based on their chemical structure, including organophosphates, organochlorines, carbamates, neonicotinoids, and pyrethroids [1,2]. Each group has unique properties that determine its application and environmental impact [3]. Parathion-ethyl (EP), a potent organophosphorus pesticide (OPP), is widely used to control a variety of agricultural and household pests, including aphids, capsids, leaf miners, sawflies, and weevils [4]. The chemical formula for EP is C_10_H_14_NO_5_PS, and it is known by various names, such as O, O-Diethyl-O(p-nitrophenyl) phosphorothioate, parathion-ethyl, di-EP, and locally as “Folidol”. It is commonly applied to crops such as vegetables, string beans, and rice [5]. While EP is highly effective in controlling pests, its use has raised significant health and environmental concerns due to its toxicity. The United States Environmental Protection Agency (US EPA) has classified EP as a highly hazardous chemical under the Acute Toxicity category [6].

Additionally, it is considered a carcinogen due to its potential to cause long-term adverse health effects. The toxic mechanism of EP is linked to its action as a potent acetylcholinesterase (AChE) inhibitor. AChE is a critical enzyme in the nervous system, responsible for breaking down acetylcholine, a neurotransmitter essential for proper neural communication [5,6,7]. When EP inhibits AChE, acetylcholine accumulates at synaptic junctions, disrupting regular neural activity. This leads to neurological dysfunction in pests and poses severe health risks to humans. Acute exposure to EP can cause symptoms such as impaired concentration, reduced motor and cognitive function, anxiety, confusion, tremors, seizures, and, in severe cases, death [8]. Chronic, low-level exposure to EP remains less understood but is particularly concerning for vulnerable populations, such as young children and fetuses, whose nervous systems are still developing. These groups are at higher risk due to their rapid brain growth and limited ability to metabolize and detoxify chemicals. Animal studies have further emphasized the harmful effects of organophosphate exposure during critical developmental periods, highlighting the need for caution in using such pesticides. The widespread use of EP has contaminated food, water, and soil, creating significant risks for human health and ecosystems. The persistence of EP in the environment, coupled with its high toxicity, makes it one of the primary causes of pesticide poisoning. As a result, the use of EP has been restricted or even discontinued in many regions worldwide [8,9]. Regulatory bodies like the European Union have set strict guidelines to limit exposure to this harmful pesticide. The European Union Pesticides Database has established the maximum residue limit (MRL) for EP at 0.01 ppm, reflecting growing concerns about its toxicity. Similarly, the US EPA has defined MRLs for various organophosphates, ranging from 0.01 ppm to 3 ppm, depending on the specific pesticide and the type of food [10]. For EP, it is critical to ensure compliance with these limits to minimize the risks to public health. Long-term exposure to EP has also been linked to a range of severe medical conditions. Research has suggested that prolonged exposure may contribute to the development of diseases such as Parkinson’s disease, asthma, attention deficit hyperactivity disorder (ADHD), and certain types of cancer. These medical conditions further underscore the importance of regulating EP residues in food, water, and the environment [11]. Therefore, effectively monitoring and detecting EP residues is essential to protect public health.

Conventional methods for detecting EP and other organophosphorus pesticides include techniques such as gas chromatography (GC), high-performance liquid chromatography (HPLC), and capillary electrophoresis (CE). While these methods are known for their high sensitivity, selectivity, and reproducibility, they also have significant drawbacks. For instance, sample preparation can be time-consuming and labor-intensive, instrumentation can be expensive, and these methods often require toxic and volatile solvents [5,6,7,8,9,10,11]. Additionally, GC is unsuitable for polar or thermally labile compounds, and HPLC requires multiple processing steps, making the overall process more complex and costly. In contrast, electrochemical detection has emerged as a promising alternative for monitoring EP residues. Electrochemical sensors are known for their simplicity, cost-effectiveness, high sensitivity, and stability [12,13,14]. Nitroaromatic compounds are electrochemically active, and their reducible nitro group allows for detecting various analytes at low concentrations [15,16]. Electrochemical sensors have become increasingly popular due to their adaptability and ability to provide reliable and real-time detection of analytes [17].

Recent advancements in nanomaterials have further enhanced the performance of these sensors. Nanomaterial-modified electrodes offer a larger active surface area, enhanced nano-porous structures, and high loading capacity, all of which contribute to improved sensitivity and accuracy in detection [18]. The efficiency of an electrochemical sensor largely depends on the properties of the working electrode surface. For the effective detection of EP, it is crucial to modify the electrode with a catalyst that exhibits excellent electrochemical properties. This ensures the sensor interacts effectively with the analyte, enabling precise and sensitive detection [19]. In previous studies, Huang et al. reported the electrochemical detection of alkyl parathion, achieving a limit of detection (LOD) of 0.172 µM using a Pd/MWCNTs-modified electrode for the determination of methyl and ethyl parathion [2]. Similarly, Wei et al. developed an Al-doped mesoporous cellular foam-modified electrode for the detection of parathion pesticides, also achieving a LOD of 0.0172 µM [3]. These findings highlight the effectiveness of modified electrode materials in enhancing the sensitivity and selectivity of pesticide detection. Based on the literature, we used Zn_2_SnO_4_ to detect EP.

Current developments in zinc stannate (Zn_2_SnO_4_) in electrode materials and surface modifications have significantly improved the performance of electrochemical sensors designed for analyte detection [20]. Zn_2_SnO_4_ is a versatile spinel-type material with a three-dimensional cubic structure, and it has gained significant attention in various applications, such as lithium-ion batteries, gas sensors, and photocatalysis. The crystal structure of Zn_2_SnO_4_ belongs to the Fd^3^m space group, and the material is composed of zinc (Zn^2+^) and oxygen (O^2−^) ions occupying tetrahedral and octahedral sites, respectively. At the same time, stannum (Sn^4+^) resides within oxygen octahedra. This unique configuration, combined with oxygen vacancies, enhances the adsorption of target analytes, making Zn_2_SnO_4_ an excellent material for electrochemical sensing applications [21,22,23]. Sol–gel offers good mixing but needs calcination and may cause agglomeration. Co-precipitation and combustion are scalable but lack precision and require high temperatures. The hydrothermal method excels, with high crystallinity, morphology control, and purity and is eco-friendly, optimizing Zn_2_SnO_4_ performance [24]. The hydrothermal method is favored for its simplicity and ability to produce high-purity materials with controlled nanostructures [25,26].

Zn_2_SnO_4_ nanocomposites have been explored for detecting hazardous compounds, but their application in electrochemical sensing of nitroaromatic compounds like EP remains underexplored. This presents an opportunity for further research.

In this study, zinc stannate (Zn_2_SnO_4_) nanopowder was synthesized using a simple hydrothermal method, demonstrating enhanced electrochemical properties for the sensitive and selective detection of EP under ambient conditions. The sensor showed lower detection limits and good reproducibility, making it ideal for real-time monitoring of EP in environmental and agricultural samples. Zn_2_SnO_4_ is a multifunctional metal oxide semiconductor (MOS) material known for its high electron mobility, lightweight nature, and strong thermal and chemical stability. Furthermore, Zn_2_SnO_4_ nanostructures enhance electrochemical sensing with increased active surface area, active sites, and efficient mass/electron transport. The material’s superior performance offers great promise for detecting hazardous substances like EP, contributing to food safety and public health protection.

## 2. Experimental Sections

### 2.1. Chemical and Reagents

All chemicals, including zinc nitrate hexahydrate, tin (IV) chloride pentahydrate, cetyltrimethylammonium bromide, n-butylamine, potassium ferrocyanide (K_4_[Fe(CN)_6_]·3H_2_O, potassium ferricyanide (K_3_[Fe(CN)_6_]), and the analyte, EP, were of analytical grade and obtained from Sigma Aldrich, Alfa Aesar and Showa Chemical Industry Co., Ltd. and were used as received without additional purification. Sodium phosphate dibasic (Na_2_HPO_4_, ≥99%) and sodium dihydrogen phosphate (NaH_2_PO_4_, ≥99%) were employed to prepare a 0.1 M phosphate buffer (PB) solution, which served as the supporting electrolyte for all electrochemical experiments. Distilled water (DI) was used to make the solutions. Prior to use, glassware was thoroughly cleaned with ethanol to ensure contaminant-free experiments. A measure of 5 mM ferricyanide [Fe(CN)_6_]^3−/4−^ solution was prepared by adding potassium ferrocyanide (K_4_[Fe(CN)_6_]·3H_2_O) and potassium ferricyanide (K_3_[Fe(CN)_6_]) in 0.1 M KCl. Further details regarding electrolyte preparation and instrumentation are available in the Supplementary Information.

### 2.2. Instrumentation

The Zn_2_SnO_4_ nanomaterial was characterized using various analytical techniques. The phase composition was determined through X-ray diffraction (XRD) analysis using a Bruker 2D Phaser instrument. The crystal structure was visualized with the help of Vesta software (version 3.90.5a). Fourier-transform infrared (FT-IR) spectroscopy was performed using a PerkinElmer spectrometer within the range of 400–4000 cm^−1^ to identify functional groups and molecular structures. These characterization techniques provided insights into the physical properties of the synthesized material. The electrochemical performance was evaluated using a CHI 1211c electrochemical workstation, employing cyclic voltammetry (CV) in a conventional three-electrode setup. A glassy carbon electrode (GCE) with a surface area of 0.072 cm^2^ served as the working electrode, while a saturated Ag/AgCl electrode and a platinum wire functioned as the reference and counter electrodes, respectively.

### 2.3. Synthesis of Electrocatalyst Zn_2_SnO_4_/SnO_2_ by Hydrothermal Method

**Figure 1:**
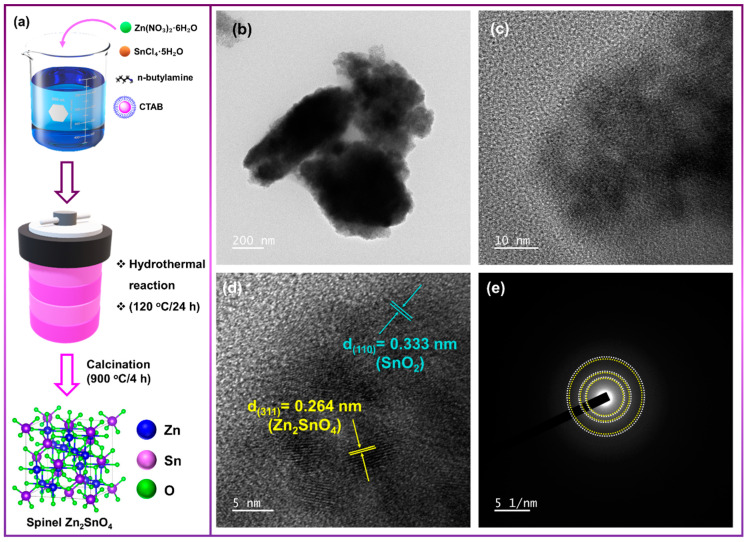
(**a**) Schematic illustration of the synthesis of Zn_2_SnO_4_/SnO_2_ nanoparticles. (**b**–**d**) Different magnification images of Zn_2_SnO_4_/SnO_2_ heterostructures. (**d**) Lattice fringes and (**e**) SAED patterns of Zn_2_SnO_4_/SnO_2_ heterostructures.

A 0.1 M solution of zinc nitrate hexahydrate (Zn(NO_3_)_2_·6H_2_O) and a 0.05 M solution of tin(IV) chloride pentahydrate (SnCl_4_·5H_2_O) were prepared and stirred at room temperature until completely dissolved in 150 mL of DI water. To this mixture, 5 mM of cetyltrimethylammonium bromide (CTAB) and 1 g of n-butylamine were added under continuous stirring to form a homogeneous solution. This solution was transferred to a Teflon-lined autoclave and subjected to hydrothermal treatment at 120 °C for 24 h. The resultant product was washed three times with deionized water and ethanol. Then, the washed product was then dried at 90 °C for 12 h and calcined at 900 °C for 4 h to obtain Zn_2_SnO_4_/SnO_2_ as the electrocatalyst (Figure 1a).

### 2.4. Electrode Surface Modifications

Drop casting is a simple and efficient method for fabricating modified electrodes using suitable electrode materials. In this process, 2 mg of the synthesized Zn_2_SnO_4_/SnO_2_ nanoparticles were dispersed in 1 mL of deionized water through sonication for 15 min to ensure a uniform suspension. Prior to modification, a glassy carbon electrode (GCE) was polished with 0.05 μm alumina powder to remove any surface impurities and achieve a smooth surface. A measure of 4–10 μL of the Zn_2_SnO_4_ nanoparticle suspension was carefully pipetted from the prepared suspension onto the polished GCE. Upon application, the droplet experienced a surface tension gradient that induced an outward convective flow, referred to as Marangoni flow, driven by interfacial forces. As the inward Marangoni flow exceeded the outward capillary flow, the solute concentrated at the center of the droplet, promoting the adsorption of Zn_2_SnO_4_/SnO_2_ particles onto the electrode surface. The modified electrode was dried at 80 °C for 3 min to ensure adhesion and prepared for subsequent electrochemical experiments.

### 2.5. Optimization of the Detection System

CV and differential pulse voltammetry (DPV) techniques were used to detect EP using a three-electrode system, in which modified Zn_2_SnO_4_/SnO_2_/GCE was the working electrode and platinum wire and Ag/AgCl electrodes served as reference and counter electrodes, respectively. The DPV measurements were performed using a CHI1211C electrochemical workstation with a step potential of 0.004 V, a pulse amplitude of 0.05 V, and a pulse width of 0.025 s. The pulse period was set to 0.05 s, with a quiet time of 2 s, and the potential range spanned from 0.4 V to −1.2 V The electrochemical response was recorded in a PB at different pH levels. The scan rate and concentration dependence were analyzed to evaluate sensor performance.

## 3. Results and Discussions

### 3.1. Transmission Electron Microscopy (TEM) Analysis

To examine the Zn_2_SnO_4_/SnO_2_ sample’s morphology, transmission electron microscope (TEM) analysis has been characterized. The Zn_2_SnO_4_/SnO_2_ sample’s TEM images are shown in Figure 1b–d. Examining the sample with a TEM image at a greater magnification reveals that it comprises monodisperse microspheres. This confirms that the nanoparticles comprise aggregated nanoparticles with diameters ranging from approximately ≈5 to 10 nanometers. Figure 1d displays a TEM image of the Zn_2_SnO_4_/SnO_2_. The successful formation of the Zn_2_SnO_4_/SnO_2_ heterojunction is indicated by the HRTEM images of the sample, which show that the d-spacing of ≈0.264 nm plane corresponds to the (311) crystal planes of Zn_2_SnO_4_/SnO_2_ [27] (Figure 1d) and that the d-spacing of the ≈0.333 nm plane corresponds to the (110) crystal planes of SnO_2_ [28]. The SAED pattern of the Zn_2_SnO_4_/SnO_2_ heterostructure, as shown in Figure 1e, indicates the polycrystalline nature the sample and the SAED pattern’s diffraction rings can be linked to (311) and (220) for Zn_2_SnO_4_/SnO_2_ and (110) and (200) for SnO_2_ [29].

### 3.2. X-Ray Diffraction (XRD) Analysis

**Figure 2:**
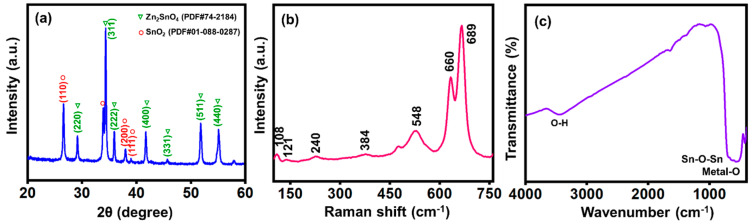
(**a**) The XRD pattern of cubic Zn_2_SnO_4_/SnO_2_ heterostructure. (**b**) Raman and (**c**) FTIR spectrum of Zn_2_SnO_4_/SnO_2_ heterostructure.

The structural properties of the Zn_2_SnO_4_/SnO_2_ sample were analyzed using X-ray diffraction Fi(XRD), and the results are shown in Figure 2a. The XRD pattern of the Zn_2_SnO_4_/SnO_2_ sample exhibited distinct peaks consistent with the reference code 74-2184 [22], corresponding to a cubic crystal system and space group *Fd-3m*. The identified peaks for Zn_2_SnO_4_ were located at 2theta values of 29°, 34°, 36°, 42°, 53°, and 56°, corresponding to the (220), (311), (222), (400), (511), and (440) planes, confirming the formation of Zn_2_SnO_4_. In addition to the Zn_2_SnO_4_ peaks, there were two or three weak peaks corresponding to SnO_2_ at around 27°, 38°, and 53°, which are associated with the (110), (200), and (111) planes, respectively [22]. These peaks suggest a minor presence of SnO_2_ in the sample, confirming that Zn_2_SnO_4_ was synthesized with a slight impurity or incomplete conversion of the SnO_2_ precursor.

### 3.3. Raman Spectroscopy Analysis

Raman spectroscopy was employed to provide further insights into the structural features of Zn_2_SnO_4_/SnO_2_ shown in Figure 2b, and it was allotted to T_2u_, B_1g_, F_2g_, E_g_, A_2u_, B_2g_, and A_1g_ symmetries. The peaks observed at 108 cm^−1^ and 121 cm^−1^ were associated with T_2u_ and B_1g_ modes, respectively. Raman modes observed at 240 cm^−1^ and 384 cm^−1^ correspond to the E(LO) phonon and F_2g_ modes. A peak at 548 cm^−1^ is attributed to the internal vibrations of the oxygen tetrahedron. The band at 660 cm^−1^ arises from the Zn-O bonds symmetric stretching in the SnO_4_ tetrahedra (inverse Zn_2_SnO_4_). Additionally, the peak at 689 cm^−1^ corresponds to the A_2u_ mode. The existence of the Zn_2_SnO_4_/SnO_2_ occurs solely in a heterostructure, as demonstrated by all of these Raman modes [22].

### 3.4. Fourier-Transform Infrared Spectroscopy (FTIR) Analysis

The Zn_2_SnO_4_/SnO_2_ samples were analyzed using FTIR spectroscopy, with the spectrum shown in Figure 2c. The Zn_2_SnO_4_/SnO_2_ sample exhibited characteristic stretching vibration at 586 cm^−1^ which is attributed to the vibration of the metal–oxygen bond. Additionally, a band at 880 cm^−1^ corresponds to the stretching vibration of the Sn–O bond. A broad stretching vibration at 3305 cm^−1^ is attributed to the O–H bond stretching, indicating the presence of absorbed water molecules on the sample’s surface, further confirmed by a peak at 1646 cm^−1^. These Raman and FTIR results confirm the presence of both Zn_2_SnO_4_ and a few peaks of SnO_2_ in the sample [22,23].

### 3.5. Electro-Catalytic Behaviors of Zn_2_SnO_4_/SnO_2_ Through Redox Probe

**Figure 3:**
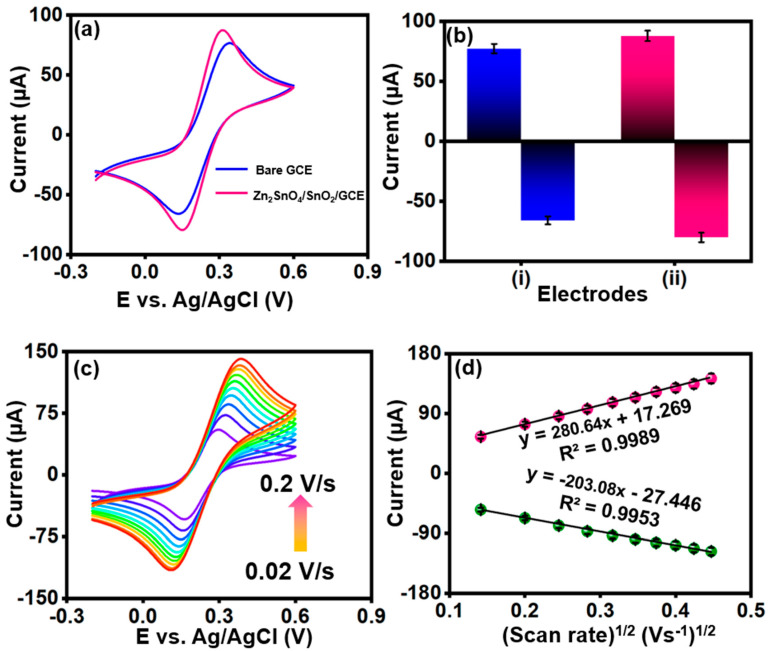
(**a**,**b**) CV profiles of bare GCE and Zn_2_SnO_4_/SnO_2_/GCE were shown in a solution containing 5 mM [Fe(CN)_6_]^3−/4−^ and 0.1 M KCl. (**c**) CV measurements of Zn_2_SnO_4_/SnO_2_/GCE at different scan rates (0.02 Vs^−1^ to 0.22 Vs^−1^) in 5 mM [Fe(CN)_6_]^3−/4−^ and 0.1 M KCl and linear plot of peak current vs. square root of the scan rates (**d**).

The electrochemical performance of the Zn_2_SnO_4_/SnO_2_-modified electrode was assessed through electrochemical impedance spectroscopy (EIS) in a 0.1 M KCl solution containing 5 mM [Fe(CN)_6_]^3−/4−^. The Nyquist plot and the corresponding Randles circuit model shown in Figure S1 demonstrate the charge transfer characteristics at the electrode-electrolyte interface. The unmodified GCE exhibited a higher charge transfer resistance (R_ct_) of approximately 55.99 Ω, whereas the Zn_2_SnO_4_/SnO_2_-modified electrode displayed a significantly lower R_ct_ of 38.13 Ω, indicating enhanced electrical conductivity and improved electron transfer kinetics. The reduction in R_ct_ suggests that Zn_2_SnO_4_/SnO_2_ facilitates better charge transport due to its increased electroactive surface area and efficient electron mobility. The electroactive behavior of the bare electrode and the modified electrode was evaluated through signal amplification in cyclic voltammetry studies conducted in 5 mM [Fe(CN)_6_]^4−^/[Fe(CN)_6_]^3−^ redox solution containing 0.1 M KCl. The CV profile, depicted in Figure 3a, has distinct redox peaks for each modified electrode, resulting from the reversible electron transfer of the [Fe(CN)_6_]^4−^/[Fe(CN)_6_]^3−^ redox pair. The bare GCE showed inferior redox performance of I_pa_ = 75.93 µA at E_pa_ = 0.34 V and I_pc_ = −62.86 µA at E_pc_ = 0.13 V compared to the modified electrode, attributed to the lack of additional pathways for efficient electron transfer that would enhance the reversible redox reaction. Upon modification, the GCE surface and the ionic aqueous environment within the interlayer region considerably improved electrochemical activity, as demonstrated by the notable increase in current of I_pa_ = 91.28 µA at E_pa_ = 0.31 V and I_pc_ = −76.35 µA at E_pc_ = 0.137 V for the Zn_2_SnO_4_/SnO_2_/GCE (Figure 3b). The electrochemical behavior of Zn_2_SnO_4_/SnO_2_/GCE was investigated by varying the scan rate from 0.02 Vs^−1^ to 0.2 Vs^−1^ using cyclic voltammetry in the same electrolyte used for other modified electrodes. As shown in Figure 3c, the redox peak currents increased proportionally with the applied square root of the scan rate, indicating a diffusion-controlled electrochemical process. The corresponding linear plots for the anodic and cathodic peak currents versus the square root of the scan rate are presented in Figure 3d.

These high correlation coefficients confirm the linear relationship between the peak currents and the square root of the scan rate, reflecting efficient electron transfer kinetics at the Zn_2_SnO_4_/SnO_2_/GCE surface. Using the obtained data and the Randles–Sevcik equation (Equation (1)), the electroactive surface area of the Zn_2_SnO_4_/SnO_2_/GCE was calculated, further demonstrating its enhanced electrochemical performance. *I_p_* represents the redox peak current response in the above equation, while *n* denotes the number of electrons involved in the electrochemical reaction.

(1) $$I_{p}=2.69\times 10^{5}n^{2/3}{AD}^{1/2}v^{1/2}C$$

The parameter *A* corresponds to the electroactive surface area, *D* is the diffusion coefficient (considered constant), *C* represents the electrolyte concentration, and *ν* is the applied scan rate. Using Equation (1), the calculated electroactive surface area for the Zn_2_SnO_4_/SnO_2_/GCE was found to be 0.076 cm^2^, highlighting the enhanced electron transfer properties due to surface modification.

### 3.6. Electrochemical Behaviors of Zn_2_SnO_4_/SnO_2_/GCE Towards EP Detection

**Figure 4:**
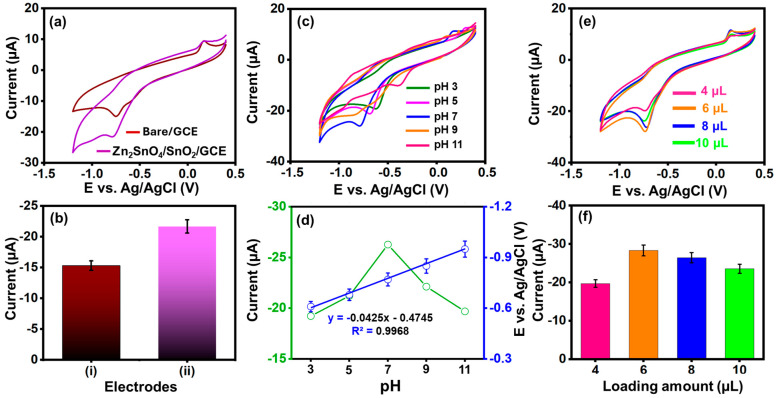
(**a**) CV profiles of bare and Zn_2_SnO_4_-modified electrodes in 150 µM EP and (**b**) bar diagrams of peak current show an improvement in current response with the (i) bare/GCE and (ii) Zn_2_SnO_4_/SnO_2_/GCE-modified electrode. (**c**) CV profiles of Zn_2_SnO_4_/SnO_2_/GCE-modified electrode at different pH values and (**d**) the plots of peak current (green) and peak potential (blue) vs. pH. (**e**) CV profiles of Zn_2_SnO_4_/SnO_2_/GCE with different loading amounts (4, 6, 8, and 10 μL) and (**f**) bar diagrams of peak current reveal that the 6 μL loading amount produces the maximum current response, while 4, 8, and 10 μL result in lower current responses. All experiments were performed in 0.1 M phosphate buffer (pH 7.0) at a scan rate of 0.05 Vs^−1^, except pH study.

The electrochemical behavior of EP was systematically investigated using CV at bare GCE and Zn_2_SnO_4_/SnO_2_/GCE. The CV measurements were conducted in 0.1 M PB solution containing EP at a scan rate of 0.05 Vs^−1^. Figure 4a shows the CV responses for both bare and Zn_2_SnO_4_/SnO_2_/GCE. The results highlight the comparative performance of the electrodes in the presence of EP, illustrating differences in their electrochemical responses. The CV profiles, shown in Figure 4b, display the peak current responses for the bare GCE and Zn_2_SnO_4_/SnO_2_/GCE in the presence of EP, recorded within the potential window of 0.4 V to −1.2 V at a scan rate of 0.05 Vs^−1^. The bare GCE exhibits a peak current of −15.11 μA, while the Zn_2_SnO_4_/SnO_2_/GCE shows a significantly higher peak current of −21.87 μA. This indicates that the bare GCE demonstrates limited electron transfer between the electrode and electrolyte, resulting in a lower reduction peak current. In contrast, the Zn_2_SnO_4_/SnO_2_/GCE substantially enhances the current response, suggesting that the modification improves the electrochemical performance. This improvement can be attributed to the increased active surface area provided by the Zn_2_SnO_4_/SnO_2_/GCE nanostructures, facilitating better interaction with the electrolyte. Additionally, the high conductivity of Zn_2_SnO_4_/SnO_2_/GCE promotes more efficient electron transfer, further enhancing the electrochemical response. Thus, the Zn_2_SnO_4_/SnO_2_/GCE modification significantly improves the electrode’s ability to detect EP, as evidenced by the higher reduction peak current compared to the bare GCE. Figure 4b presents a bar diagram comparing the reduction peak currents for the bare GCE and Zn_2_SnO_4_/SnO_2_/GCE, further illustrating the enhanced performance of the Zn_2_SnO_4_/SnO_2_/GCE.

### 3.7. Effect of Supporting Electrolyte

The choice of supporting electrolytes plays a crucial role in determining the electrochemical performance of Zn_2_SnO_4_/SnO_2_/GCE-modified electrodes for EP detection. For the effect of pH, a study was conducted using Zn_2_SnO_4_/SnO_2_/GCE in the presence of 150 μM EP, with the supporting electrolyte adjusted to pH levels ranging from 3 to 11 at a fixed scan rate of 0.05 Vs^−1^. As shown in Figure 4c, the reduction peak current increased progressively as the pH of the medium increased from 3 to 11, reaching its maximum at a neutral pH of 7. Beyond this, at pH 11, the peak current diminished. These findings indicate that the shift in the reduction peak as a function of pH is typically due to the protonation or deprotonation of electroactive species. More protons are available in acidic conditions, which can facilitate electron transfer, shifting the peak to more positive potentials. Conversely, fewer protons are available in basic conditions, making the reduction process more difficult and shifting the peak to more negative potentials. The results demonstrate that a neutral pH of 7 provides the most favorable environment for the reduction process, offering the highest current response and stable electrochemical performance. Figure 4d plots the reduction peak currents and potentials at different pH values. Based on these observations, pH 7 was the optimal condition for the supporting electrolyte in subsequent electrochemical applications. As a result, variations in the electrochemical reaction were observed through changes in the CV response potential and current, which directly influenced the electron transfer kinetics. The relationship between current and potential as a function of pH is illustrated in Figure 4d. By correlation with a Nernst equation, theoretical values are almost closer to our experimental slope value of −0.042 V pH^−1^, which accounts for the involvement of an equal no. of protons in electrochemical reactions and establishes the relationship between peak potential and pH. The corresponding Nernst equation is applied for a response involving m protons and n electrons. As the pH increases, the peak potential shifts negatively, confirming that the electrochemical process involves proton loss. The corresponding linear equation is given by y = −0.0425pH − 0.4745, with a correlation coefficient of R^2^ = 0.9968.

### 3.8. Effect of Catalyst Loading Amount

The influence of varying the loading amounts of Zn_2_SnO_4_/SnO_2_/GCE on the modified GCE was examined in the presence of 150 μM EP. The CV profiles for different loading amounts of Zn_2_SnO_4_/SnO_2_/GCE ranging from 4 to 10 μL are presented in Figure 4e. As the loading increased, the reduction peak current gradually enhanced, indicating improved electrochemical activity. However, further increasing the loading amount beyond 6 μL, up to 10 μL, resulted in a decline in peak current. The electrochemical response increases with catalyst loading up to 6 μL, beyond which a decrease in peak current was observed due to particle aggregation, which hinders electron transfer between the electrode surface and the electrolyte. The highest reduction peak current of I_pc_ = −21.87 μA was observed with an optimal loading of 6 μL (Figure 4f). Thus, a 6 μL loading amount was selected as the optimum condition for subsequent electrochemical studies.

### 3.9. Impact of Different Concentration and Scan Rates

**Figure 5:**
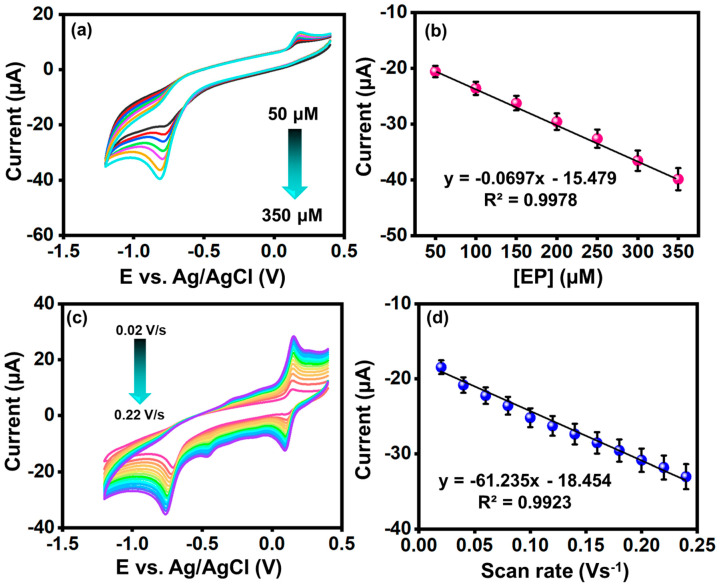
(**a**) CV curves of Zn_2_SnO_4_/SnO_2_/GCE at different concentrations of EP show an increase in peak currents with higher concentrations and (**b**) the calibration plot of current versus concentration display a clear linear relationship, ensuring reliable quantification. (**c**) CV curves at various scan rates for 150 µM EP in pH 7.0 show a proportional increase in peak current with the scan rate and (**d**) the linear plot of current (µA) versus scan rate (Vs^−1^).

The electrochemical performance of Zn_2_SnO_4_/SnO_2_/GCE was evaluated through CV in 0.1 M phosphate buffer (pH 7.0) with incremental additions of EP. Figure 5a illustrates the CV profiles recorded at a fixed scan rate of 0.05 Vs^−1^ as the concentration of EP was increased sequentially between 50 μM and 350 μM. The results reveal a consistent rise in the cathodic peak current with each addition of EP, demonstrating the effective interaction between the analyte and the modified electrode surface. Additionally, the unique properties of the Zn_2_SnO_4_/SnO_2_/GCE heterostructure enhance the cathodic peak current by reducing capacitance and preventing fouling effects. This antifouling behavior stems from the synergistic interactions between the Zn_2_SnO_4_/SnO_2_/GCE heterostructure and the electrode surface, ensuring a stable and reproducible performance. The efficacy of the Zn_2_SnO_4_/SnO_2_/GCE modification in accurately detecting EP is further demonstrated in Figure 5b, which presents a clear linear relationship between the EP concentration and the corresponding cathodic peak current. This linearity is described by a strong correlation coefficient and the regression equation provided Equation (2).

(2) $$I_{pc}(µA)=-0.0697\left[EP\right](\mu M)-15.479,\;R^{2}=0.9978$$

Furthermore, in observing the kinetic behavior of Zn_2_SnO_4_/SnO_2_/GCE, CV analysis was performed in 0.1 M phosphate buffer (pH 7.0) by varying the scan rate from 0.02 Vs^−1^ to 0.2 Vs^−1^, as illustrated in Figure 5c. The CV profiles show a notable increase in reduction peak current with an increase in scan rate, highlighting the electrode’s ability to facilitate rapid electron transfer. The kinetic analysis indicates that the Zn_2_SnO_4_/SnO_2_/GCE follows a surface-controlled process. The direct proportionality between the cathodic peak current and the scan rate, a hallmark of surface-dominated electron transfer mechanisms, supports this observation. The unique properties of Zn_2_SnO_4_/SnO_2_-modified GCE, such as its enhanced electron transport capabilities, high active surface area, and efficient ion exchange, contribute to its superior electrochemical performance. The linear relationship between the cathodic peak current and the scan rate is represented in Figure 5d. The regression equation and corresponding correlation coefficient value for the Zn_2_SnO_4_/SnO_2_/GCE are expressed in Equation (3).

(3) $$I_{pc}\left(µA\right)=-61.235\;v({Vs}^{-1})-18.454,\;\;R^{2}=0.9923$$

**Scheme 1:**
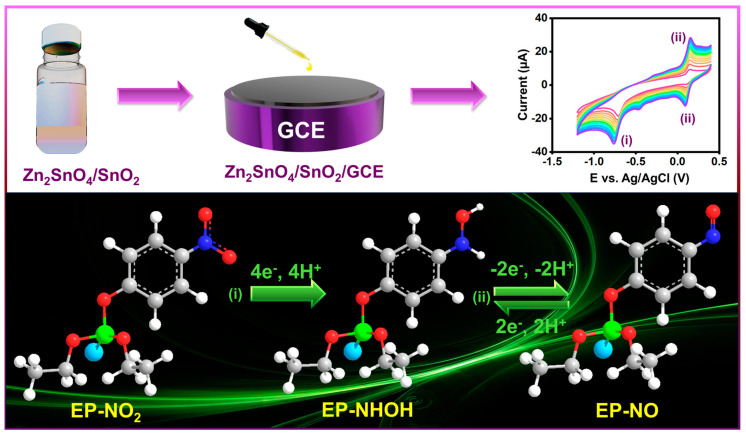
The electrochemical reduction/oxidation reaction of EP at Zn_2_SnO_4_/SnO_2_/GCE.

Scheme 1 shows that a sharp cathodic peak (E_pc_(i)) at −0.71 V was found in the first cycle, which conforms with the literature reports. This peak was caused by the reduction in the nitro group of EP (EP-NO_2_), which resulted in its hydroxylamine derivatives (EP-NHOH). In the first cycle’s reverse part, a redox peak (ii) is associated with the nitroso group (EP-NO) formed by the oxidation of EP-NHOH. Furthermore, this two-electron-transfer process, which can be reversed, formed a peak reduction (ii) throughout the subsequent CV potential scan.

### 3.10. Differential Pulse Voltammetry (DPV) Analysis

**Figure 6:**
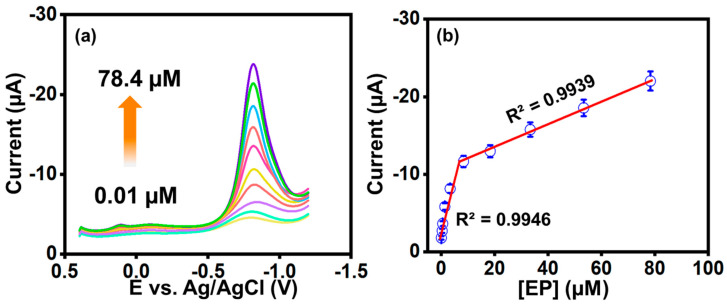
(**a**) DPV curves of Zn_2_SnO_4_/SnO_2_/GCE with the concentration range of EP from **0.01 to 78.4** µM in a 0.1 M PB solution and (**b**) the linear plot of peak current vs. EP concentration.

The electrochemical detection of EP was investigated using differential pulse voltammetry (DPV) for its enhanced selectivity and sensitivity over CV. Electrochemical analysis was performed at low concentrations to ensure precise detection of EP. A 0.01–78.4 µM concentration range of EP was analyzed using a Zn_2_SnO_4_/SnO_2_/GCE in a 0.1 M PB solution with a three-electrode system. The DPV results showed a gradual increase in the reduction peak current with increasing EP concentration, indicating enhanced response due to the effective interaction of the analyte with the modified electrode surface. These DPV responses are depicted in Figure 6a, with the corresponding calibration plots for lower and higher concentration ranges, as shown in Figure 6b, to determine the limit of detection (LOD). The LOD was calculated using Equation (4).

(4) LOD=3S/σ

Here, *S* represents the standard deviation from the DPV responses and *σ* is the slope derived from the calibration plot. The calculated LOD for EP detection using Zn_2_SnO_4_/SnO_2_/GCE was 0.0059 µM. The performance of this study was compared with previously reported electrochemical sensors, focusing on the modified electrodes, LOD, and linear detection ranges, as summarized in Table 1. These results highlight the effectiveness of the Zn_2_SnO_4_/SnO_2_/GCE in the sensitive and precise detection of EP.

### 3.11. Selectivity, Repeatability, and Reproducibility Study

To assess sensor selectivity, the DPV method was performed in the presence of common interfering compounds including Parathion methyl, quercetin, 4-nitrophenol, theobromine, 2-nitrophenol, roxarsone, and mercury ions, which are similar to EP compound (Figure S2). A concentration of 100 μM EP along with 200 μM of the interferents was used. The modified electrode Zn_2_SnO_4_/SnO_2_/GCE exhibited excellent selectivity toward the target analyte, even in the presence of structurally similar or commonly interfering substances. The higher current response for the analyte suggests strong surface interaction and favorable electron transfer dynamics. This indicates that the sensor can distinguish the analyte with high specificity, making it a reliable tool for selective detection. The durability of the fabricated Zn_2_SnO_4_/SnO_2_/GCE for EP detection was evaluated through repeatability and reproducibility analyses. The repeatability study was carried out using the CV technique, with four consecutive measurements conducted in the presence of 20 µM EP (Figure S3a). The results demonstrated consistent and stable responses across all measurements, confirming the Zn_2_SnO_4_/SnO_2_/GCE reliability for 20 μM EP detection with the RSD values of ±3.79%. The study used three independently fabricated Zn_2_SnO_4_/SnO_2_/GCE under identical experimental conditions to assess reproducibility. The results revealed minimal variation in the reduction in current responses across the electrodes, highlighting the robustness and reproducibility of the sensor fabrication process. This indicates that the Zn_2_SnO_4_/SnO_2_/GCE provides consistent performance for EP detection, regardless of the electrode batch (Figure S3b). The stability of the fabricated Zn_2_SnO_4_/SnO_2_/GCE was assessed using cyclic voltammetry in 0.1 M PB (pH 7.0) containing 150 µM ethyl parathion at a scan rate of 0.05 Vs^−1^. The electrode maintained a stable current response over 40 successive cycles, suggesting strong durability and surface integrity. Approximately 75% of the original peak current was preserved, reflecting low signal drift and minimal material loss during repeated usage (Figure S3c). These results confirm the reliable repeatability, reproducibility, and cyclic stability of the Zn_2_SnO_4_/SnO_2_-modified electrode for the electrochemical detection of EP.

### 3.12. Real Sample Analysis

The electrochemical performance of Zn_2_SnO_4_/SnO_2_/GCE was further evaluated using real samples, such as river water and carrot samples, to assess its applicability in detecting EP in complex matrices. These real samples were pre-treated, first as the river water samples were obtained from the Xindian River in Taiwan and then pretreated using Whatman filter paper to eliminate particulate matter. Electrochemical testing of the un-spiked samples exhibited no measurable current, indicating the absence of ethyl parathion. To assess the sensor’s performance in real matrices, standard solutions of ethyl parathion were introduced into both rivers. Following this spiking, clear and concentration-dependent electrochemical responses were recorded, confirming the sensor’s effectiveness in detecting ethyl parathion within complex water samples. On the other hand, the chopped carrot sample was soaked in deionized (DI) water at a mass-to-water ratio of 1:5 for 30–60 min to allow extraction. After soaking, the mixture was centrifuged at 8000–9000 rpm for 10–15 min to obtain a pure extract. The extract was used for further analysis. The detection was carried out by progressively adding known amounts of EP to the samples and measuring the electrochemical response using Zn_2_SnO_4_/SnO_2_/GCE. The results, shown in Table S1 indicate that as the concentration of EP increased, a corresponding increase in the cathodic current was observed, demonstrating the electrode’s sensitivity and ability to detect EP in complex samples. The analysis was repeated for two consecutive additions, with stable and reproducible results observed in all real sample analyses.

## 4. Conclusions

In this study, Zn_2_SnO_4_/SnO_2_/GCE was successfully developed and applied for the electrochemical detection of EP. The unique structural and electrochemical properties of Zn_2_SnO_4_/SnO_2_/GCE, including its high active surface area, enhanced conductivity, and catalytic activity, facilitated the efficient detection of EP with higher sensitivity and a low detection limit of 0.0059 µM. The electrode demonstrated excellent repeatability, stability, and selectivity, even in the presence of potential interferents. Furthermore, the Zn_2_SnO_4_/SnO_2_/GCE was tested on real samples such as river water and carrot extracts, where it showed reliable recovery rates, highlighting its potential for practical applications in environmental and agricultural monitoring. The results confirm that the Zn_2_SnO_4_/SnO_2_/GCE sensor is a promising tool for rapid, sensitive, and selective detection of pesticide residues, contributing to the advancement of electrochemical sensing technologies for environmental safety and food quality control.

## Figures and Tables

**Table 1:** Comparison of analytical results for the detection of EP with the proposed electrode.

Materials	Linear Ranges (µM)	LOD (µM)	Ref.
CS-FeO	0.34–4.46	0.187 µM	[4]
AA-P3TAA	0.5–100	0.5 µM	[6]
Silver nanoparticle-modified electrode	0.0412–7.90	0.0412 µM	[30]
Carbon nanoparticles and halloysite nanoclay modified CPE	0.0012–4.81	0.00038 µM	[31]
Zn_2_SnO_4_/SnO_2_/GCE	0.01–8.7; 8.7–78.4	0.0059 µM	This work

## Data Availability

Data sharing not applicable.

## Supplementary Materials

The following supporting information can be downloaded at: https://www.mdpi.com/article/10.3390/s25092837/s1, Figure S1: EIS of bare GCE, Zn_2_SnO_4_/SnO_2_ modified electrode in 0.1 M KCl and 5 mM [Fe(CN)_6_]^3−,4−^; Figure S2: Interfering analysis of Zn_2_SnO_4_/SnO_2_ modified electrodes in 0.1 M PB in the presence of 20 µM (1) EP with the presence of co-interfering compounds such as (2) Parathion methyl, (3) quercetin, (4) 4-nitrophenol, (5) theobromine, (6) 2-nitrophenol, (7) roxarsone and (8) Hg^2+^; Figure S3: (a) Repeatability (b) reproducibility and (c) cyclic stability analysis of Zn_2_SnO_4_/SnO_2_ modified electrodes in 0.1 M PB in the presence of 20 µM EP; Table S1: Recovery ranges of EP in spiked samples.
