# Supplementary material for: Hydrothermal Synthesis of Zinc Stannate Nanoparticles for the Electrochemical Detection of Organophosphate Pesticide—Parathion-Ethyl
Source: Sensors (Basel). 2025 Apr 30;25(9):2837. doi: 10.3390/s25092837 (PMC12074467; doi:10.3390/s25092837)
Supplement: Supplementary file 1 [file sensors-25-02837-s001.zip › sensors-3559733-supplementary.pdf]

Supporting information

Hydrothermal Synthesis of Zinc Stannate Nanoparticles for the Electrochemical Detection of Organophosphate Pesticide: Parathion-ethyl

Loganathan Vagismathi,[a] *Sea-Fue Wang,*[a,*]

[a]Department of Materials and Mineral Resources Engineering, National Taipei University of Technology, Taipei 106, Taiwan.

Corresponding author:

***Dr. Sea-Fue Wang:** sfwang@ntut.edu.tw

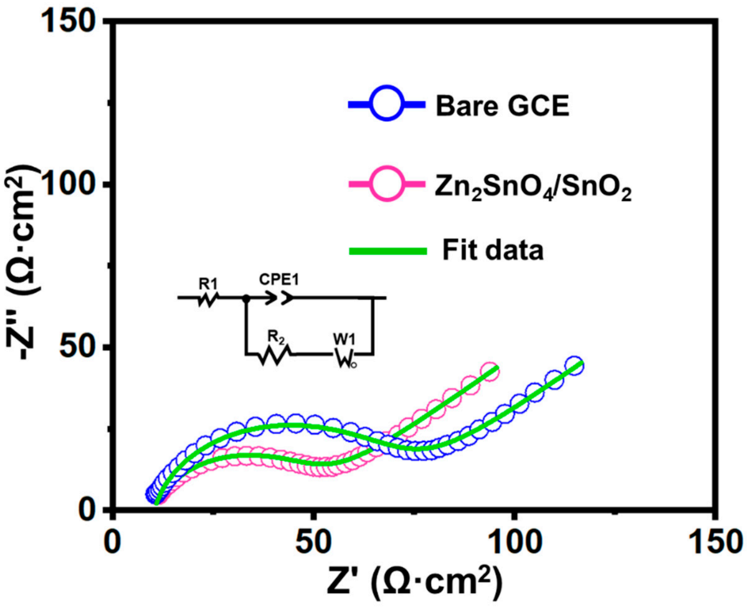

Figure S1. EIS of bare GCE, Zn_2SnO_4/SnO_2 modified electrode in 0.1 M KCl and 5 mM $[Fe(CN)_6]^{3-,4-}$.

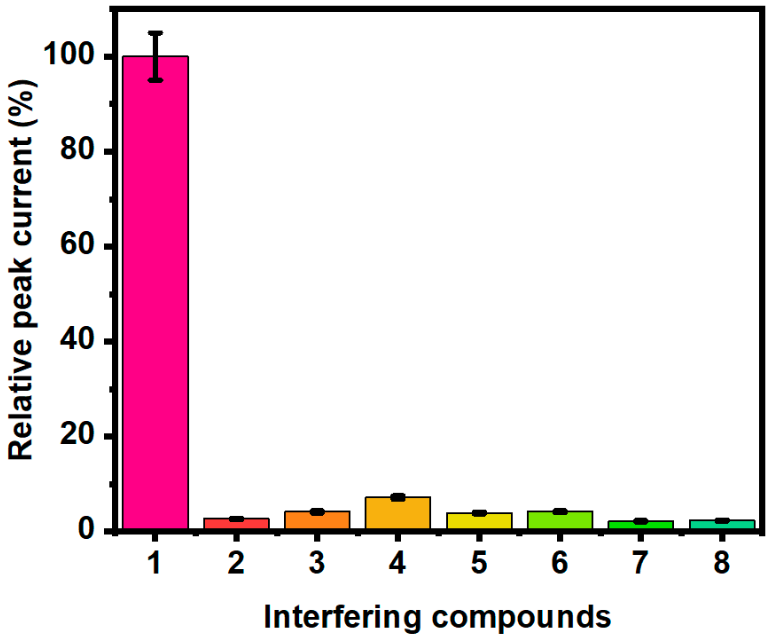

Figure S2. Interfering analysis of Zn_2SnO_4/SnO_2 modified electrodes in 0.1 M PB in the presence of (1) EP with co-interfering compounds such as (2) Parathion-methyl, (3) quercetin, (4) 4-nitrophenol, (5) theobromine, (6) 2-nitrophenol, (7) roxarsone and (8) Hg^{2+}.

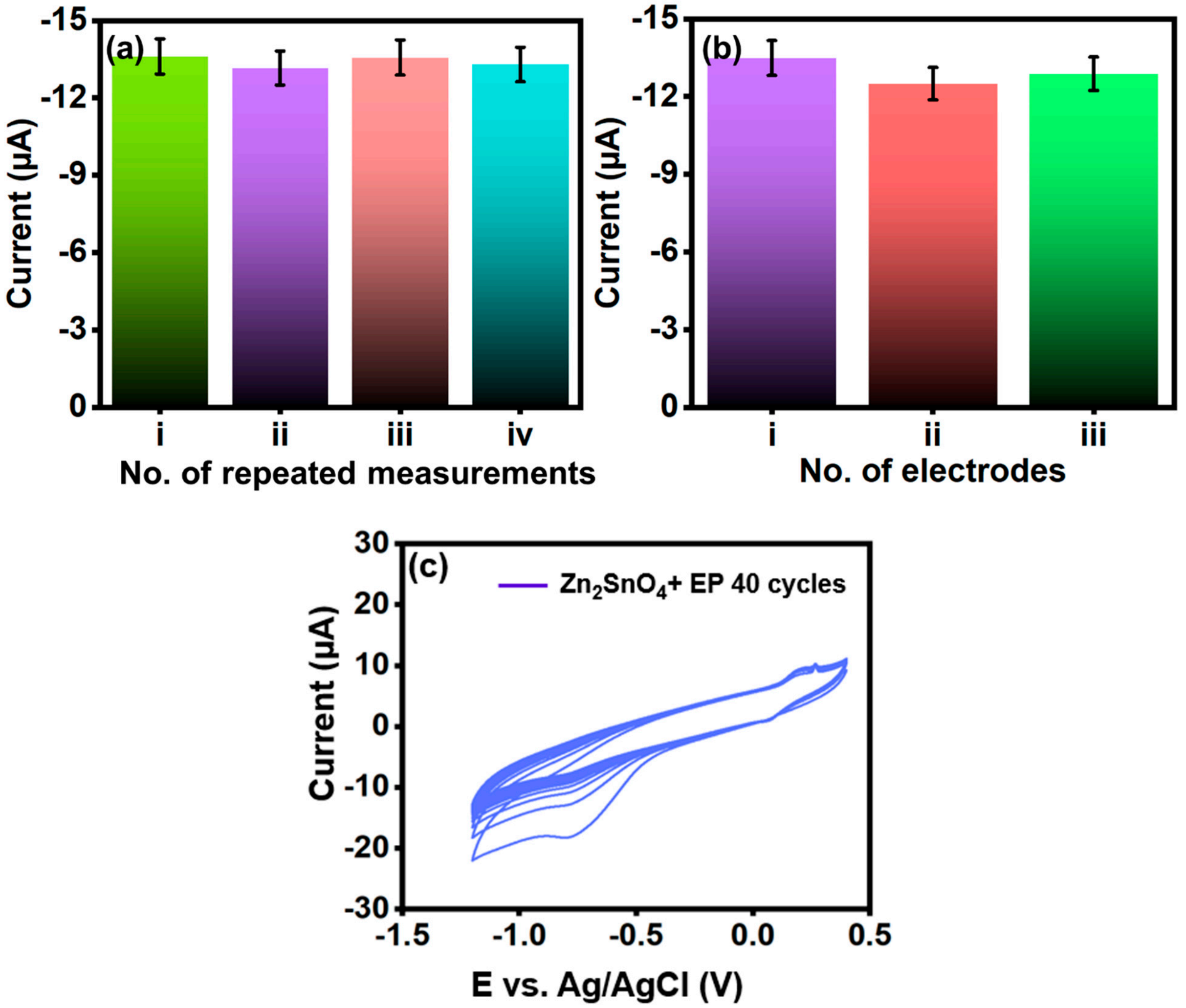

Figure S3. (a) Repeatability **(b)** reproducibility and **(c)** cyclic stability analysis of Zn_2SnO_4/SnO_2 modified electrodes in 0.1 M PB in the presence of 20 μM EP.

Table S1. Recovery ranges of EP in spiked samples.

Samples	Added (µM)	Found (µM)	Recovery (%) ±n=3
River water	0	-	-
	5	4.89	97.8±0.012
	10	9.92	99.2±0.478
Carrot	0	-	-
	5	4.97	99.4±0.083
	10	9.95	99.5±0.56
